# Impact of climate change on adipose-derived stem cells: A molecular and histological study

**DOI:** 10.1016/j.joclim.2024.100367

**Published:** 2024-11-27

**Authors:** Saeed Motesaddi Zarandi, Rasoul Yarahmadi, Rasul Nasiri, Mohammad Bayat, Hossein Nasiri, Abdollah Amini, Mohammad Esmaeil Motlagh, Hassan Rasoulzadeh

**Affiliations:** a Department of Environmental Health Engineering, School of Public Health and Safety, Shahid Beheshti University of Medical Sciences, Tehran, Iran; b Air Pollution Research Center, Department of Occupational Health Engineering, School of Public Health, Iran University of Medical Sciences, Tehran, Iran; c Air Pollution Research Center, Iran University of Medical Sciences, Tehran, Iran; d Price Institute of Surgical Research, University of Louisville, and Noveratech LLC, Louisville, Kentucky, USA; e Faculty of Geography, University of Tehran, Tehran, Iran; f Associate Professor of Biology and Anatomical Sciences Department, School of Medicine, Shahid Beheshti University of Medical Sciences, Tehran, Iran; g Department of Pediatrics, School of Medicine, Ahvaz Jondishapour University of Medical Sciences, Ahvaz, Iran; h Department of Environmental Health Engineering, Maragheh of Medical Sciences, Tehran, Iran

**Keywords:** Climate change, Health impacts, Adipose-derived stem cells

## Abstract

**Background:**

Climate change, particularly temperature and humidity fluctuations, can affect biological systems. This study specifically investigates the impact of these two key aspects of climate change on adipose-derived stem cells (ADSCs) as a pilot study and starting point for further examinations into the biological effects of climate change-associated conditions.

**Methods:**

One-month-old male rats were kept for 4 months (equal to a 10-year climatic period) in 4 groups and exposed to conditions based on climatic data from Tehran's synoptic stations. Rats in the control group were exposed to conditions based on climatic data from 1991, and groups 2–4 were exposed to conditions based on climatic data from 1991 to 2000, 2001–2010, and 2011–2020, respectively. Monthly temperature, humidity, and day/night cycle averages were selected for animal exposure. After exposure, fat-derived stem cells were taken from each subject and assays assessing reactive oxygen species (ROS), cell viability and proliferation, and apoptosis were performed.

**Results:**

From 1991 to 2020, Tehran experienced an average temperature increase of 1.5 °C and a 15 % decrease in average humidity, conditions which when replicated in rodent models were associated with increased rates of ROS and caspase-3 expression, a reduction in Ki-67 antigen expression and in the duration of the life of cells, and an increase in the rate of apoptosis, such that apoptosis in ADSCs reached 25.51 %.

**Conclusion:**

The study demonstrates that environmental conditions similar to those from climate change are associated with significant changes in ADSCs and emphasizes the need for further research to understand their impact on health.

## Highlights

• Climate change exhibits a myriad of impacts on society and the environment.
• The uncharted territory of health implications from climate change on living organisms in vivo awaits exploration.
• Our study pioneers the investigation into the effects of climate change on adipose-derived stem cells (ADSCs) in vivo.
• Our research breaks new ground in understanding these impacts, unveiling significant direct effects of climate change on ADSCs.

## Graphical abstract

**Figure:**
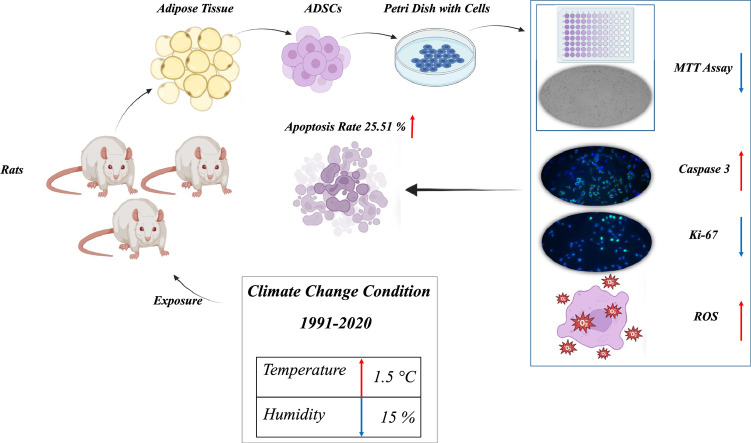

## 1 Introduction

Today we are witnessing changes in climate at a historically fast pace, and their effects are quickly becoming manifest on societies and the environment globally. Concentrations of greenhouse gases such as CO_2_ and CH_4_ have increased dramatically since 1950, leading to increased global warming [1]. This has become an escalating trend, as human activities generate greater amounts of greenhouse gas emissions, which result in extreme weather conditions occurring with increasing intensity [[2], [3], [4]]. Between 1880 and 2012, average temperatures rose globally by 0.85 °C (range = 0.65–1.06 °C) [2,4]. The United Nations Intergovernmental Panel on Climate Change (IPCC) in its fifth assessment report on climate change (IPCC, 2014) has predicted that based on Representative Concentration Scenarios (RCPs) RCP2.6, RCP4.5, RCP6, and RCP8.5, increases in the average global temperature of 0.3–1.7, 1.1–2.6, 1.4–3.1, and 2.5–4.8 °C, respectively, compared to the years 1986–2005 will occur during the years 2081–2100 [1,5]. Each scenario reflects different levels of greenhouse gas emissions and their potential impacts on global temperatures. As these increases occur, humankind will encounter warmer and more extreme weather [4,6,7]. Consequently, the world must direct its attention to the challenges presented by climate change in the current century [4,8]. Urban heat islands cause cities to be more greatly affected by temperature increases than smaller towns [1,9]. Subsequently, the effects of climate change are expected to intensify, ultimately threatening urban residents with serious problems [1,10]. Tehran, as the capital of Iran with the largest population and highest population density, faces all the aforementioned challenges. Therefore, it was chosen as the model for this study due to easier access to more complete and comprehensive climate data, allowing us to effectively utilize its climate conditions for our research.

The range of climate change impacts is broad and varied. Recent research has assessed how climate change has affected public health [11,12], women's health [[13], [14], [15]], mental health [16], eco-anxiety [17], health and food security [[18], [19], [20]], social inequalities [21], infectious diseases such as malaria [22,23], arbovirus diseases like dengue [24,25], and numerous parasitic and viral diseases, such as Japanese encephalitis [26], Rift Valley Fever [27], human African trypanosomiasis [28], and leishmaniasis [29]. Other research has explored how temperature changes affect reproduction [[30], [31], [32], [33]]. Studies have reported that sudden seasonal rise and fall in temperature as well as gradual temperature changes in the genitalia expose the testes and sperm to temperature stress, resulting in increased sperm DNA fragmentation, weakened sperm mitochondria function and motility, and sperm lipid peroxidation [[30], [31], [32], [33]]. Using case studies involving a variety of diseases and climate conditions, scientists increasingly have identified and modeled how climate change impacts various aspects of health [[34], [35], [36]].

Adipose-derived stem cells (ADSCs) are an abundant, readily available population of multipotent progenitor cells found in adipose tissue, making them an accessible source for translational clinical research [37]. Compared to bone marrow-derived mesenchymal stem cells (BM-MSCs), ADSCs have a significantly higher stem cell density in tissue (5 % versus 0.01 %) and can be obtained through a less invasive and painful procedure [38]. These advantages, along with their potential for differentiation and relevant paracrine effects, make ADSCs an ideal model for investigating the effect of ambient conditions on stem cell health [39]. Various assays can be employed to examine cellular responses, including the MTT (3-(4,5-Dimethylthiazol-2-yl)-2,5-Diphenyltetrazolium Bromide) assay for cell viability and proliferation [40], reactive oxygen species (ROS) analysis for oxidative stress levels [41], Ki-67 staining for cellular proliferation [42], Caspase-3 activity measurement for apoptotic pathways, and apoptosis assays to determine cell death mechanisms [43]. To date, however, how climate change affects health at the cellular level, particularly the cellular perturbations caused by temperature and humidity fluctuations seen in climate change scenarios, has not been studied. Therefore, the current research aimed to address this issue by investigating how temperature and humidity fluctuations related to climate change affect ADSCs, serving as a foundation for future studies on the potential health impacts of climate change. While climate change encompasses a broad range of factors, this study specifically focused on the effects of two critical aspects—temperature and humidity fluctuations—on ADSCs, utilizing climate data from Tehran and based on a previously published protocol [1]. These meteorologic factors were chosen as they are key components in climate change scenarios that can have measurable impacts on biological systems.

## 2 Materials and methods

### 2.1 Study design

The protocol and pilot designed for the current study were used to investigate in vivo how ADSCs are affected by conditions experienced as consequences of changes in climate. The exposures differed among study groups with variable climatic-data-based conditions.

### 2.2 Study groups with variable climate-based exposure conditions

This study examined 24 one-month-old male Wistar rats divided into 4 groups and housed in chambers with dimensions of 60 cm × 60 cm × 70 cm for long-term exposure to specific climatic conditions. One-month-old male rats were kept for 4 months (functionally equal to a 10-year climatic period) in 4 groups and exposed to conditions based on climatic data from Tehran's synoptic stations, as described below.

All animals were maintained in full compliance with animal welfare and care protocols and in accordance with the ethical guidelines of Shahid Beheshti University of Medical Sciences and the Guide for the Care and Use of Laboratory Animals (NIH Publication, revised 1996). All experimental procedures were conducted following approval by the Shahid Beheshti University of Medical Sciences Ethics Committee (Ethical code: IR. SBMU.PHNS. REC.1400.114).

Climatic data from the Tehran metropolitan area for the years 1991–2020 were used to create exposure conditions for rats in each of four groups. Group 1, the control group, was exposed to conditions derived from climate data from 1991. Group 2 was exposed to conditions corresponding with the years 1991–2000, Group 3 from 2001 to 2010, and Group 4 from 2011 to 2020. Monthly temperature, humidity, and day/night cycle averages were selected for animal exposure (for more details, refer to [1]).

Table 1 provides descriptive statistics for temperature and humidity in Tehran as a monthly average during the years 1991–2020. Temperature increased and humidity decreased during these years; from 1991 to 2020, the monthly average temperature increased by almost 1.5 °C, and the monthly average humidity decreased by 10–15 % relative to its initial value [44].

**Table 1:** Statistical description of temperature and humidity in Tehran during 1991–2020.

**Groups**	**Parameter**	**25th percentile**	**75th percentile**	**SD**	**CV**	**24-hour Mean**	**Mean night**	**Mean day**	**Min**
**1991 (control, group one)**	Temperature °C	8.62	23.48	9.95	0.61	16.37	13.86	18.89	3.26
	Humidity %	29.19	55.27	15.97	0.36	43.83	50.30	37.37	15.97
**1991–2000 (group two)**	Temperature °C	7.90	26.14	9.44	0.56	16.84	14.35	19.34	0.56
	Humidity %	31.76	54.76	13.73	0.32	43.24	49.85	36.64	23.86
**2001–2010 (group three)**	Temperature °C	9.36	26.50	9.22	0.52	17.79	15.02	20.57	−2.45
	Humidity %	29.72	51.89	13.51	0.33	40.65	47.99	33.31	18.06
**2011–2020 (group four)**	Temperature °C	8.95	26.75	9.18	0.53	17.44	14.85	20.05	3.61
	Humidity %	23.64	47.70	13.96	0.38	36.57	43.17	29.97	14.56

*Group 1 = control.

### 2.3 Isolation, expansion, and immunophenotyping of ADSCs

At the end of four months, animals were euthanized using CO₂ inhalation, following approved ethical guidelines to minimize distress. The CO₂ flow rate was controlled to ensure a gradual increase, allowing for humane and painless euthanasia (NIH Publication, revised 1996). Approximately 3 cc of adipose tissue was extracted from the subcutaneous abdominal region of the adult rats. The extracted tissue underwent isolation, culturing, and expansion using established protocols [45]. Following this, the samples were examined for markers indicative of mesenchymal stem cells (MSCs) using flow cytometry techniques outlined in previous publication [46].

### 2.4 Cell survival test by the MTT method

ADSCs were cultured at 5 × 10^3 cell/well in a 96-well plate and then incubated for 24 h to permit cell adherence to the plate bottom. After this period of treatment, 10 μl of MTT stock solution (Carl Roth, Germany) was added to each well with 90 μl cultivation medium to check cell viability, and incubation continued for another 3 to 4 h at 37 °C. The addition of MTT stock solution causes the color of the environment to turn blue indicating formazan (a viability marker) has been produced. The plates were then removed from the incubator, and the supernatant of the cells was removed. Next, each well received one ml of DMSO to dissolve the produced crystals, and the samples were incubated for 15 min more. Finally, the researchers pipetted each plate and spectrophotometrically measured absorbance at the wavelength of 570 nm.

The percentage of cells surviving was calculated using the following formula:

Survival percentage of each treatment sample=absorption of treatment sample / absorption of the control sample × 100

### 2.5 ROS measurement method by flow cytometry

After cell separation with EDTA (Merck) and trypsin (Sigma), ADSCs were subjected to 5 mins of centrifugation with PBS at 1200 RPM and 4 °C in Eppendorf tubes.

Next, 100 μl 2′,7′–dichlorofluorescin diacetate (DCFDA) substance (CAS Number. 2044–85–1), a fluorescent probe used to detect reactive oxygen species (ROS) in cells, at a 20-μM concentration was added to the sample, and the sample was incubated for 45 min at 37 °C in the dark. Following the addition of 900 μl PBS, the sample underwent 5 min of centrifugation at 1200 RPM and a temperature of 4 °C [47].

In the last step, sample analysis was performed using a BD FACSLyric flow cytometry device with a wavelength of 495 nm.

### 2.6 Immunohistochemical testing

Cells were first fixed inside the plate with 4 % paraformaldehyde for 20 min and subsequently washed with PBS (Sigma-p4417) thrice at 5-min intervals. Next, Triton 0.3 % was added to the samples for 30 min to permeabilize membranes. The samples were then washed with PBS, and 10 % goat serum was added to them for 45 min to block the secondary antibody reaction. After the goat serum was removed, the samples were covered with primary antibody diluted (1:100) with PBS and refrigerated at a temperature of 2 °C to 8 °C for 24 h [48].

Twenty-four hours later, the samples were removed from the refrigerator and washed with PBS for four 5-min periods. Then, the samples were incubated with secondary antibody (diluted 1:150) at 37 °C for 1.5 h in darkness using an AriaTeb model incubator (Iran).

The samples were then moved into a dark room and washed three times. Twenty minutes after DAPI (sigma-D9542) was added to them, the samples were washed in PBS, and images were obtained through fluorescent photography using a microscope (Olympus Corporation, Tokyo, Japan).

### 2.7 The analysis of apoptosis by Annexin-PI method

First, 100,000 cells were poured into a microtube and brought to a volume of 500 μl with the binding buffer 1x included in the kit. One sample was used as a control without color to set up the device. Samples in microtubes were then mixed with 5 μl of Annexin FITC V and incubated at 4 °C in the dark for 15 min. Next, the tubes were centrifuged with 1 ml of binding buffer 1x at 1500 RPM for 5 min, the supernatant solution was discarded, and 500 μl of binding buffer 1x was added. At the time of reading, 3 μl of PI was added to the sample, and the reading was done using a Calibur Facs-BD flow cytometry device (Becton, Dickinson and Company, USA). Flow Jo software was used to draw the diagram, and prism5 software was used for statistical analysis [49].

### 2.8 Statistical analysis

Three biological replicates of each experiment were performed. Statistical analyses were achieved employing Prism 5.0 software package (GraphPad Software, San Diego, California, USA). One-way ANOVA followed by Tukey's multiple comparison test was used to analyze the data to determine differences between the experimental groups exposed to different temperature and humidity conditions. Statistical significance was considered as *p*-value < 0.05.

## 3 Results

### 3.1 ADSCs marker expression

Flow cytometry assessment demonstrated that ADSCs expressed cluster of differentiation (CD) stem cell markers CD34 and CD45 at levels of 0.33 % and 0.21 %, respectively. ADSCs exhibited 100 % expression of CD44 and CD105.

### 3.2 MTT assay

**Fig. 1:**
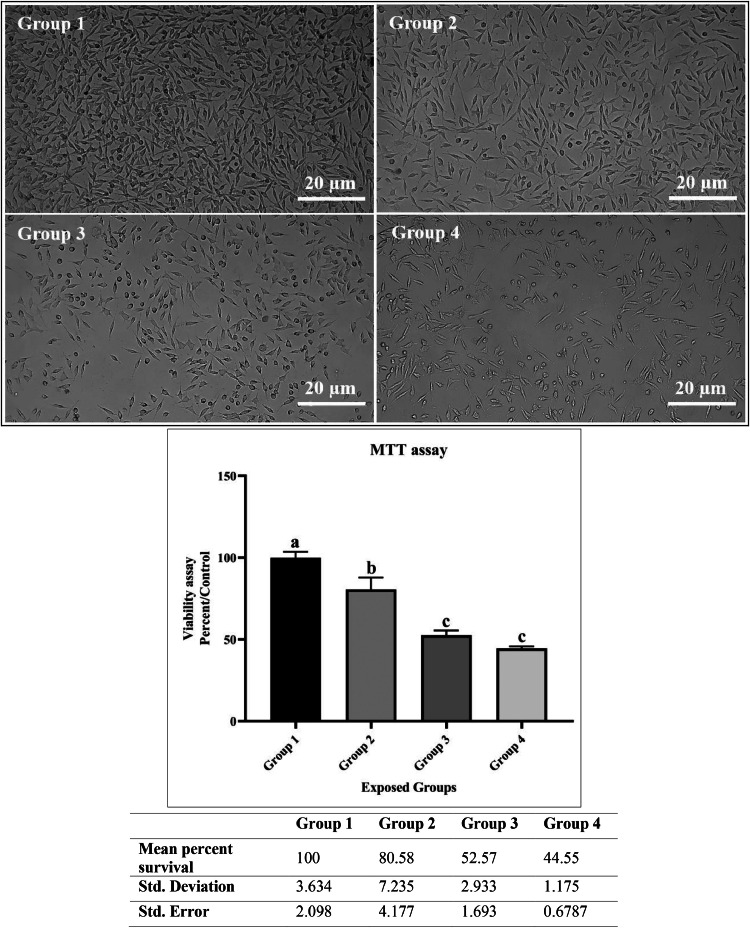
Results of the MTT test and Statistical Analysis for the 4 Studied Groups (Group 1 (1991), Group 2 (1991–2000), Group 3 (2001–2010), Group 4 (2011–2020) - reflecting Tehran's climate.). Groups with the same letter (a, b, or c) are not significantly different from each other. Groups with different letters (a, b, or c) show statistically significant differences (*P* < 0.05). For the analysis, the significance levels were defined as follows: * indicates p-value < 0.0332, ** indicates p-value < 0.0021, *** indicates p-value < 0.0002, and **** indicates p-value < 0.0001. The comparisons are as follows: Group 1 vs. Group 2 (**), Group 1 vs. Group 3 (****), Group 1 vs. Group 4 (****), Group 2 vs. Group 3 (***), Group 2 vs. Group 4 (****), and Group 3 vs. Group 4 (not significant).

The MTT assay is used to evaluate cell viability and proliferation. This assay provides insight into the overall health of the cells under varying experimental conditions and complements the apoptotic markers studied. As shown in the Fig. 1, the *MTT* results clearly shows that the control group (group 1) showed almost 100 % cell viability. The conditions of the other groups differed, however, so the cell viability for groups 2, 3, and 4 was decreased to 72-86%, 50-55%, and 43-45%, respectively. The p-value was very small at less than 0.0001, and the value of R square was 0.975. A comparison of the groups relative to each other indicated a significant difference between groups 1 and 2 and the other groups and a lack of significance in groups 3 and 4 relative to each other.

### 3.3 ROS analysis

**Fig. 2:**
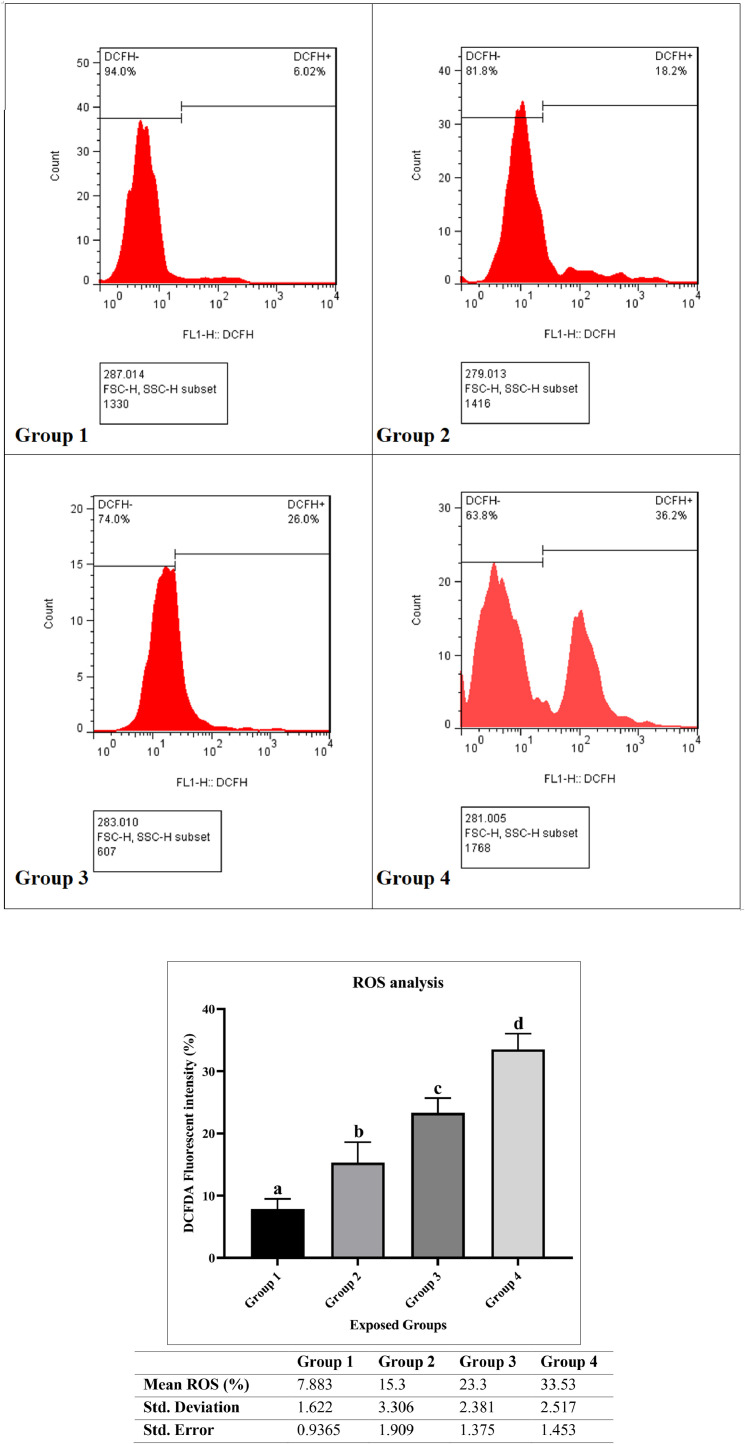
Statistical Analysis and Flow Cytometry Investigation of ROS Levels (Group 1 (1991), Group 2 (1991–2000), Group 3 (2001–2010), Group 4 (2011–2020) – reflecting Tehran's climate.). Groups labeled with different letters (a, b, c, or d) are significantly different from each other (*P* < 0.05). Each letter represents a statistically distinct group. For the analysis, the significance levels were defined as follows: * indicates p-value < 0.0332, ** indicates p-value < 0.0021, *** indicates p-value < 0.0002, and **** indicates p-value < 0.0001. The comparisons are as follows: Group 1 vs. Group 2 (*), Group 1 vs. Group 3 (***), Group 1 vs. Group 4 (****), Group 2 vs. Group 3 (*), Group 2 vs. Group 4 (****), and Group 3 vs. Group 4 (**).

The measurement of ROS is essential as these play a significant role in cellular signaling and can induce oxidative stress, leading to apoptosis. Evaluating ROS levels helps in understanding the oxidative environment's contribution to the apoptosis observed. Fig. 2 shows that the average percentages of ROS in groups 1, 2, 3, and 4 were 7.88% (range 6.02-8.98), 15.30% (11.7-16.0), 23.30% (22.4-26.0), and 33.53% (31.2-36.2) with standard deviations of 1.6, 3.3, 2.4, and 2.5, respectively. Significant differences in ROS levels among the four groups are seen, with group 4 having the highest level. The *p*-value is less than 0.0001, with an R square = 0.9551. Tukey's comparison test also revealed a significant difference in ROS levels among the studied groups, indicating an increase in ROS levels with conditions associated with increasing climate change.

### 3.4 *Caspase-3* expression assay

**Fig. 3:**
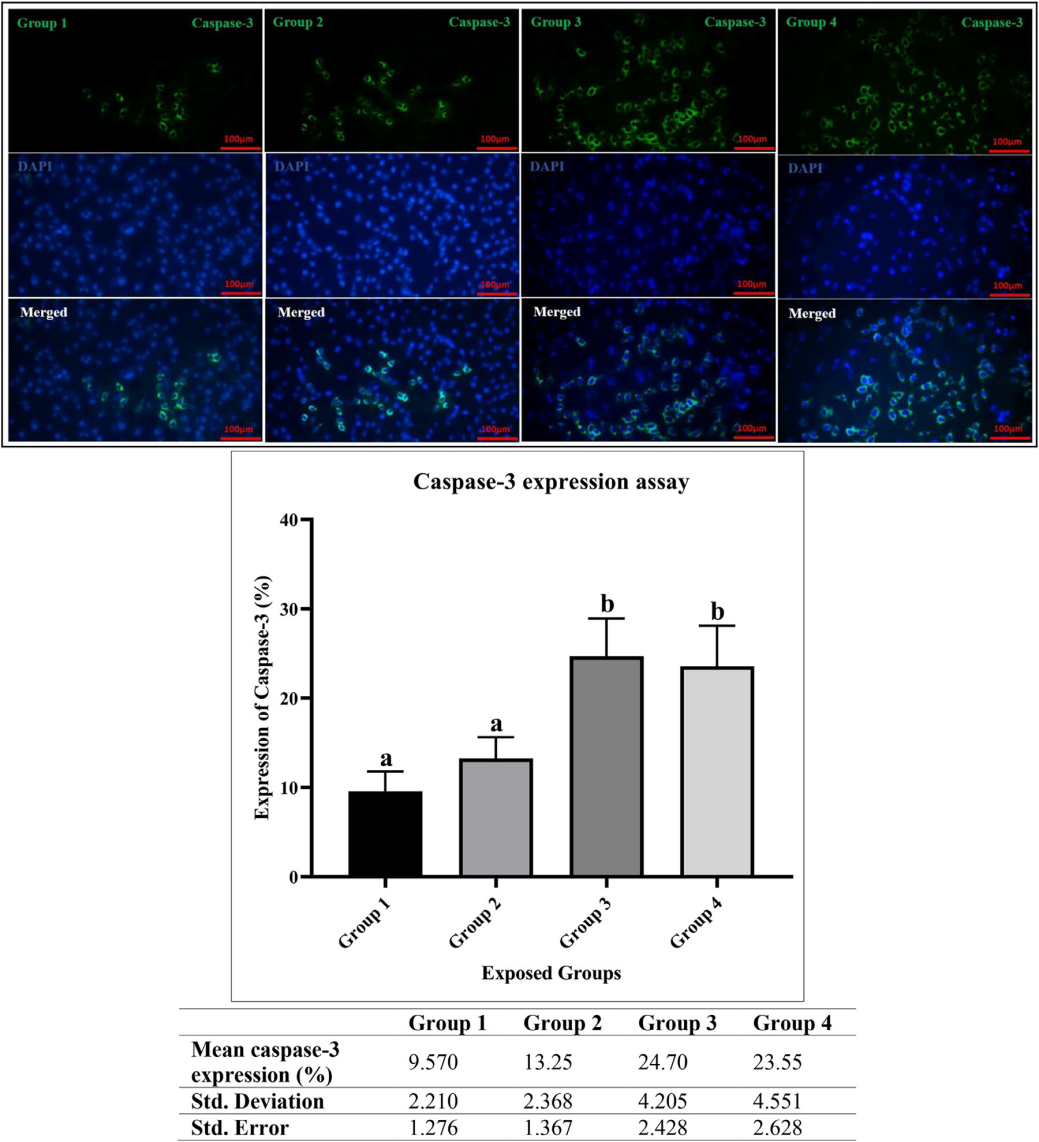
Analysis of Caspase-3 Expression Values and Images for the Studied Groups (Group 1 (1991), Group 2 (1991–2000), Group 3 (2001–2010), Group 4 (2011–2020) - reflecting Tehran's climate.). Groups labeled with the same letter (a or b) do not show a statistically significant difference from each other, while groups with different letters (a and b) are significantly different (*P* < 0.05). For the analysis, the significance levels were defined as follows: * indicates p-value < 0.0332, ** indicates p-value < 0.0021, *** indicates p-value < 0.0002, and **** indicates p-value < 0.0001. The comparisons are as follows: Group 1 vs. Group 2 (not significant), Group 1 vs. Group 3 (**), Group 1 vs. Group 4 (**), Group 2 vs. Group 3 (*), Group 2 vs. Group 4 (*), and Group 3 vs. Group 4 (not significant). The specific antibody for Caspase-3 was (cat. no. orb213644). The secondary antibody used was (rabbit, cat. no. orb688925).

Caspase-3 is a crucial executioner protease in the apoptotic pathway. Its activation is a hallmark of apoptosis, which leads to the characteristic morphological and biochemical changes associated with programmed cell death. The average caspace-3 values for groups 1, 2, 3, and 4 were 9.57 (range 7.10-11.36), 13.25 (10.72-15.41), 24.70 (20.28-28.65), and 23.55 (19.14-28.23), respectively, with standard deviation values of 2.2, 2.4, 4.2, and 4.6, respectively. The analysis of caspase-3 expression differences across groups showed that the *p*-value was less than 0.0016 with R-square = 0.8384. Tukey's test showed that there was no significant difference between groups 1 and 2, nor between groups 3 and 4; however, a significant difference was observed between groups 1 and 2 compared to groups 3 and 4, with its percentage in some subjects in groups 3 and 4 exceeding 28 % (Fig. 3).

### 3.5 Ki-67 antigen analysis

**Fig. 4:**
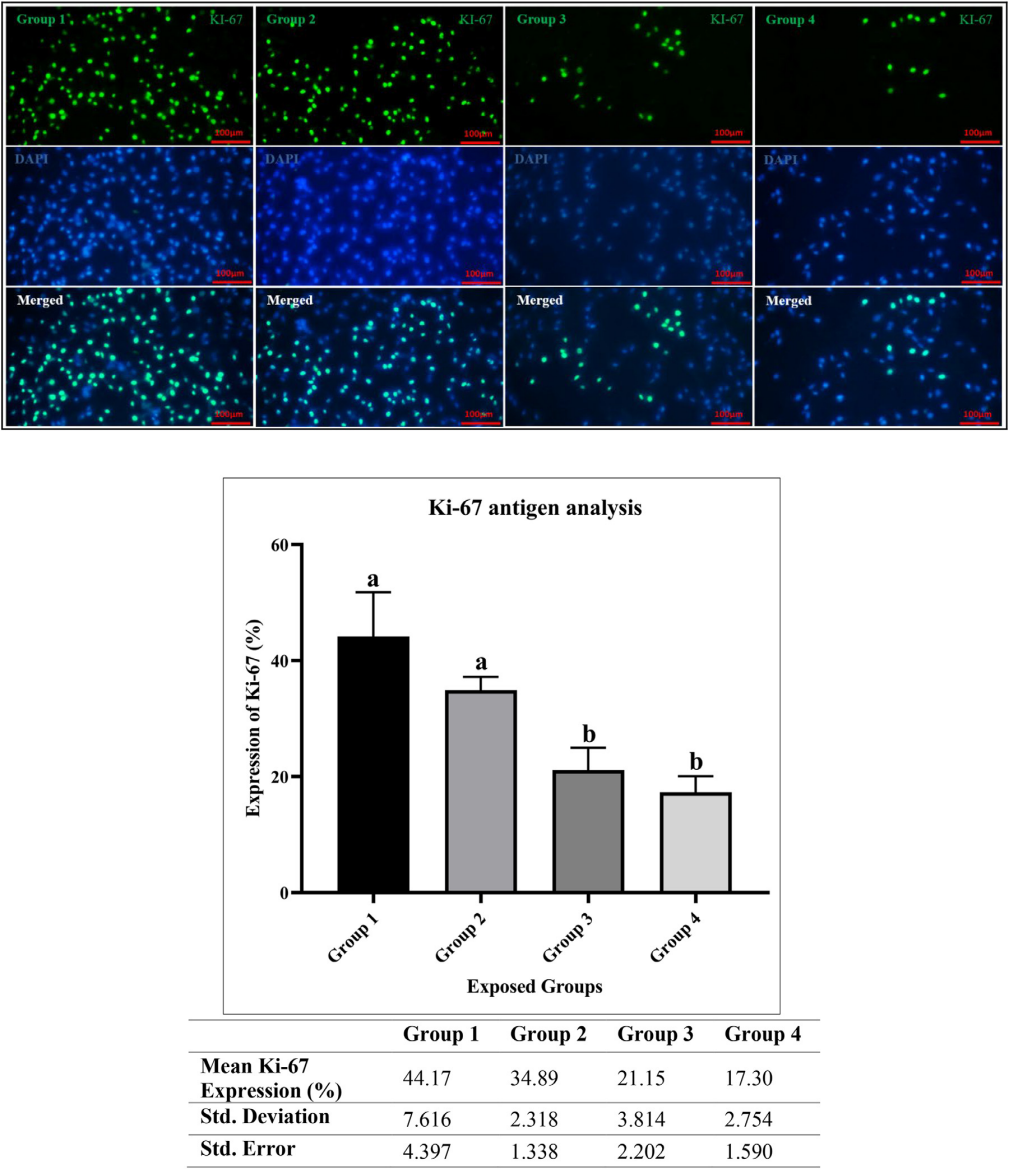
Ki-67 Antigen Expression Percentage, Statistical Description, and Immunohistochemical Images for Cell Proliferation Rate in the Studied Groups (Group 1 (1991), Group 2 (1991–2000), Group 3 (2001–2010), Group 4 (2011–2020) - reflecting Tehran's climate.). Groups labeled with the same letter (a or b) do not show a statistically significant difference from each other, while groups with different letters (a and b) are significantly different (*P* < 0.05). For the analysis, the significance levels were defined as follows: * indicates p-value < 0.0332, ** indicates p-value < 0.0021, *** indicates p-value < 0.0002, and **** indicates p-value < 0.0001. The comparisons are as follows: Group 1 vs. Group 2 (not significant), Group 1 vs. Group 3 (**), Group 1 vs. Group 4 (***), Group 2 vs. Group 3 (*), Group 2 vs. Group 4 (**), and Group 3 vs. Group 4 (not significant). The specific antibody for Ki-67 was (cat. no. orb389335). The secondary antibody used was (rabbit, cat. no. orb688925).

To evaluate the effects of oxidative stress on cellular proliferation, Ki-67 expression was analyzed. Ki-67 is an established marker for cellular proliferation, indicating cells that are actively cycling. Fig. 4 shows the immunohistochemistry results regarding cell proliferation rate obtained from the analysis of the Ki-67 antigen. The percentages of Ki-67 antigen expression for groups 1, 2, 3, and 4 showed means of 44.17 (range 35.49-49.72), 34.89 (32.91-27.39), 21.15 (17.71-25.25), and 17.30 (15.33-20.42) with standard deviations of 7.6, 2.3, 3.8, and 2.8, respectively. This shows a decrease in the cell proliferation of ADSCs associated with exposure to climate change conditions (*p* < 0.0003 and R square = 0.8904). Tukey's test showed the significance of the difference in the results obtained from the amount of Ki-67 antigen among the studied groups. Comparisons of groups 1 and 2 and groups 3 and 4 revealed nonsignificant results, which indicates that the values obtained from the Tukey's test were similar between groups 3 and 4 and groups 1 and 2. Compared to groups 1 and 2, groups 3 and 4 showed a decrease in cell proliferation for ADSCs, possibly reflecting destruction of cells or the loss of their ability to reproduce due to exposure to climatic conditions.

### 3.6 Apoptosis assay

**Fig. 5:**
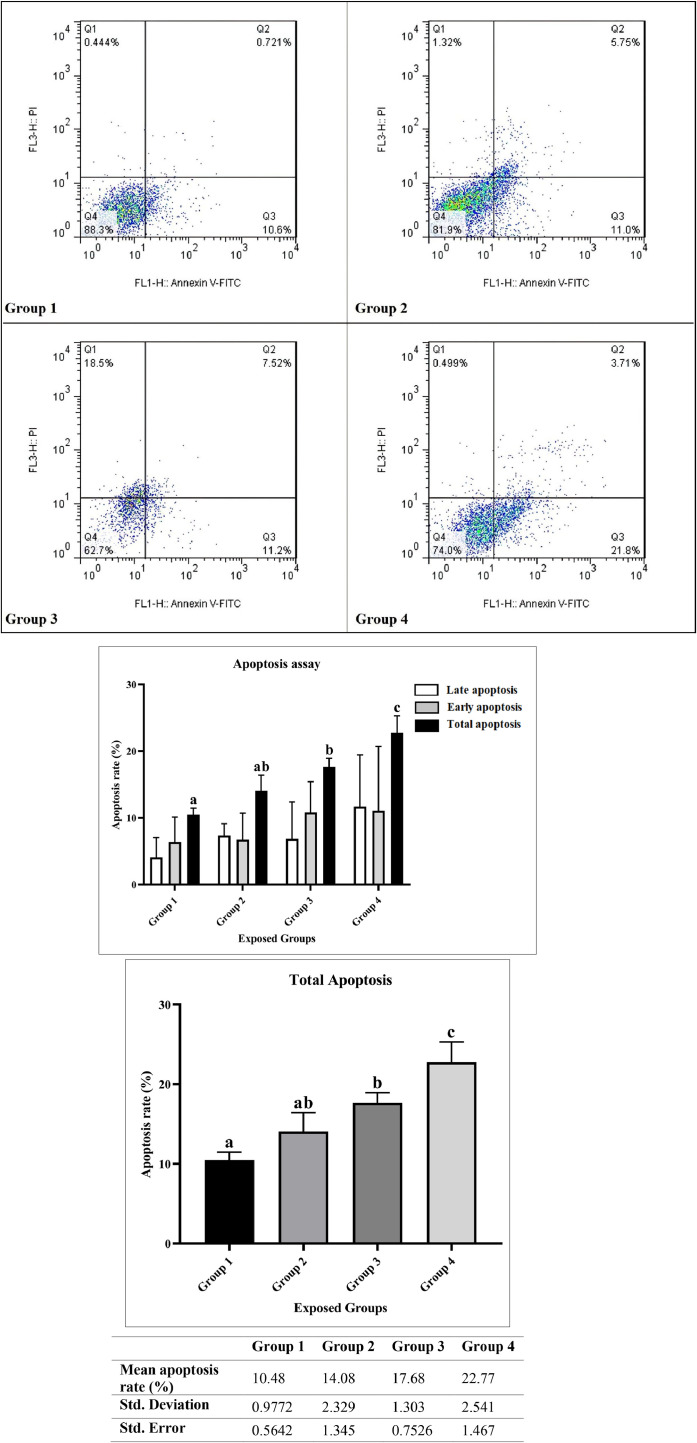
Analysis of Apoptosis Rates and Statistical Description Using the Annexin-PI Method in the Studied Groups (Group 1 (1991), Group 2 (1991–2000), Group 3 (2001–2010), Group 4 (2011–2020) - reflecting Tehran's climate). Groups labeled with the same letter (a, b, or ab) do not show a statistically significant difference from each other, while groups with different letters (a and b) are significantly different (*P* < 0.05). Groups labeled with "ab" indicate no significant difference with either group "a" or group "b". For the analysis, the significance levels were defined as follows: * indicates p-value < 0.0332, ** indicates p-value < 0.0021, *** indicates p-value < 0.0002, and **** indicates p-value < 0.0001. The comparisons are as follows: Group 1 vs. Group 2 (not significant), Group 1 vs. Group 3 (**), Group 1 vs. Group 4 (***), Group 2 vs. Group 3 (*), Group 2 vs. Group 4 (**), and Group 3 vs. Group 4 (not significant).

Fig. 5 shows the percentage values of primary apoptosis, delayed or secondary apoptosis, and total apoptosis for the studied groups. The ranges of primary apoptosis, delayed or secondary apoptosis, and total apoptosis for group 1 were 3.46–10.6, 0.721–5.95, and 9.41–11.321, respectively; for group 2, 3.2–11, 5.75–9.25, and 12.45–16.75, respectively; for group 3, 6.01–15.2, 1.02–12.1, and 16.22–18.72, respectively; and for group 4, 3.21–21.8, 3.71–19.1, and 25.51–20.49, respectively. The average percentages of apoptosis for groups 1, 2, 3, and 4 were 10.48, 14.08, 17.68, and 22.77 with standard deviations of 0.9772, 2.329, 1.303, and 2.541, respectively. The results show that group 4 experienced greater than 22 % cell apoptosis for ADSCs, indicating a significant association between climate change-derived conditions and measured apoptosis in ADSCs. As can be seen in Fig. 5, the amount of total apoptosis and delayed apoptosis increased from groups 1 to 4, respectively; group 4 and then group 2, however, experienced more primary apoptosis than group 3, though there are only small differences in the apoptosis values of groups 3 and 4. Fig. 5 also shows the slight uptick in the percentage of early apoptosis compared to delayed apoptosis in groups 2 and 4, while the amount of delayed apoptosis is higher than early apoptosis in group 3, and this difference is significant.

## 4 Discussion

The current study examined the effects of climate change-derived conditions on rat ADSCs in vivo. Previous investigations have focused mainly on how climate change impacts the occurrence of floods, droughts, fires, etc. Research dealing with aspects of how health is affected by climate change has been data-driven and based on IPCC scenarios [[50], [51], [52]]. No study, however, has examined the effects of climate change-related conditions on health *in vivo*; therefore, the present study was conducted with this gap in mind.

In the results of the MTT test for cell viability and proliferation, only the difference between groups 3 and 4 was not significant, while the differences between other groups were significant. It should be noted that group 3 had higher average temperature and humidity values compared to group 4; conversely, group 4 had relatively lower average temperature and humidity values, which suggests that the combined effect of temperature and humidity (with minor differences) was associated with a small difference in the reduction of the life of cells.

For the Caspase-3 and Ki-67 assays, no significant differences were observed between groups 3 and 4, nor between groups 1 and 2. However, it can be said that groups 1 and 2 faced climatic conditions in which temperature values increased slightly and humidity values decreased slightly for group 2, which may explain this issue to some extent. The same thing applies to groups 3 and 4; on average, the values of temperature and humidity were slightly higher for group 3 than group 4, and this may explain the closeness of the results obtained for them with Tukey's test. Another possibility may be that the effect of reducing humidity in group 4 was equal to the effect of increasing temperature in group 3, because the results of these two groups show similar cell proliferation values. While the effects of decreased humidity and increased temperature may have complex interactions, it is important to note that these variables do not necessarily produce equivalent outcomes. Therefore, further research is needed to understand the individual and combined effects of humidity and temperature on ADSCs.

In the ROS assay, significant differences were observed among all four groups, indicating that varying temperature and humidity conditions in each group substantially impacted the levels of reactive oxygen species (ROS). These significant differences suggest that changes in temperature and humidity markedly affect ROS production, which is clearly distinguished between the groups.

Regarding apoptosis, group 2 did not show significant differences from groups 1 and 3, but significant differences were observed between groups 1 and 3, and between groups 1 and 4. Additionally, the difference between groups 3 and 4 was significant. These findings indicate that the different environmental conditions may have varying impacts on apoptosis, with the reduction or increase in temperature and humidity in each group potentially influencing apoptotic responses differently.

MSCs can be isolated from various mature tissues and exhibit regenerative and immunosuppressive abilities influenced by oxygen concentration and temperature [53]. Although initially isolated from bone marrow, MSCs are called ADSCs when they are isolated from adipose tissue [54,55]. Slight changes in oxygen concentration or ambient temperature can affect stem cells and cause changes in the production of ROS, a byproduct of the mitochondrial respiratory chain, which can lead to cellular damage such as senescence, DNA damage, and apoptosis [56,57]. Physiological oxygen levels (2–8%) help reduce excessive ROS production, maintaining genetic stability, whereas higher atmospheric oxygen levels (21%) increase ROS production and genetic instability [[58], [59], [60], [61], [62]]. Studies also suggest that lowering cell temperature may reduce metabolism and mitochondrial oxygen consumption, thus limiting ROS production and offering cellular protection against oxidative damage [[63], [64], [65], [66]]. A decrease in body temperature has also been observed to prevent damage caused by ischemia in hibernating animals [67]. This protection most likely results from metabolism slowed during hibernation and the subsequent decrease in mitochondrial activity and ROS production [68,69].

Studies indicate that lowering temperature decreases cell metabolism, as shown in various cell types [[70], [71], [72], [73]]. However, reduced temperatures can also lower proliferation rates [[72], [73], [74], [75], [76]]. For instance, culturing bone marrow-derived MSCs at 32 °C reduced ROS accumulation, apoptosis, and proliferation [75]. Contrarily, when mesenchymal cells were used to establish a niche for hematopoietic stem cells, culture life and hematopoietic cell function were improved in long-term bone marrow cultured at 33 °C compared to normal culture conditions of 37 °C [77]. Similar studies have investigated the effects of temperature and environmental conditions on stem cells. Researchers in one study examined the proliferation, differentiation, accumulation of ROS, and gene expression at 35 °C and 37 °C for stem cell cultures. Their results showed that a temperature of 35 °C can lead to a decrease in the accumulation of ROS and apoptosis and an increase in the expansion and differentiation of ADSCs compared to a temperature of 37 °C. Overall, their results showed that even slight temperature changes in the culture of stem cells can profoundly affect their in vitro phenotype, yet have no impact on their proliferation rate, while possibly influencing the clinical effectiveness of cells in the body [78]. In our study, we observed a significant reduction in cell viability and increased levels of ROS and apoptosis in ADSCs exposed to higher temperatures, consistent with findings from the literature. Elevated temperatures can alter the levels of oxygen available to cells, leading to an increase in reactive oxygen species (ROS) due to enhanced mitochondrial activity and oxidative stress. Specifically, while the literature suggests that a temperature reduction may decrease ROS accumulation and apoptosis, our results indicate that extreme temperature changes, particularly elevated conditions, may lead to detrimental effects on ADSCs. This aligns with prior findings that indicate that slight temperature changes can alter stem cell behaviors, reinforcing the notion that temperature is a critical environmental factor affecting stem cell function. Thus, the observed increase in apoptosis and ROS levels in our ADSCs at elevated temperatures emphasizes the importance of maintaining physiological conditions for optimal stem cell health and functionality.

A study on primary microvascular endothelial cells (ECs) found that a 10-minute exposure to 45 °C led to irreversible damage through suppressed proliferation, increased apoptosis, delayed cell cycle, and impaired angiogenic function. Thermal stress also upregulated pro-inflammatory factors like IL-1β and IL-6, indicating systemic vascular injury potential at 45 °C [79]. In contrast, lower temperatures improved MSCs' oxidative damage indices, reduced ROS, and enhanced antioxidant levels (e.g., glutathione peroxidase and superoxide dismutase) while increasing anti-apoptotic heat shock proteins (HSP-27, −70, −90) and reducing pro-apoptotic HSP-60 [75]. Furthermore, chilling stress was shown to induce protective cell death in root stem cells, aiding stress resistance and recovery [80]. Many processes are not explicitly dependent on temperature change [81]. Generally, changes in the oxygen concentration, temperature, and mechanical stimuli activate transcription factors and downstream genes [82].

All of the mentioned studies were carried out in vitro and in a culture environment, and the effects of environmental parameters on cells were investigated for limited times. However, what is important is that environmental parameters strongly influence ROS and the expression, proliferation, and differentiation of stem cells. Most of the mentioned studies have shown that when temperature is increased, ROS and cell apoptosis are subsequently increased. As mentioned, these studies were done in vitro and in culture medium; their results, however, are in agreement with those of the present study (increases in ROS and caspase-3 expression, decreases in cell viability and Ki67 antigen expression, and an increase in the amount of apoptosis), which was conducted under completely different conditions in terms of exposure to environmental and climatic parameters. These studies evidence the gravity of investigating how conditions associated with climate change affect health, especially ADSCs, in vivo. Nevertheless, one of the principal limitations of this study lies in the novelty of its focus: to date, no research has investigated the effects of climate change on health within in vivo systems. This study thus stands as the first to explore the impact of climate-induced environmental changes specifically on adipose-derived stem cells (ADSCs). The lack of comparable studies restricts direct benchmarking of our findings, underscoring a critical gap in the current literature and the pressing need for further research. Such studies are essential to deepen our understanding of how the evolving climate may influence cellular behaviors and health outcomes in living systems.

## 5 Conclusion

The present study examined how conditions associated with climate change, precisely reflecting decades of measured meteorologic variables, affect ADSCs in vivo, aiming to shed light on a climate change effect on health that has been largely overlooked by researchers and decision-makers. Current assessments of climate change risks on health predominantly focus on immediate damages associated with climate risks, posing a challenge to incorporate long-term and direct health effects into the assessment framework. The results obtained indicate that elevated temperature and reduced relative humidity led to a decrease in MTT, while increasing ROS and Caspase-3 levels, and decreasing Ki-67 antigens, ultimately resulting in increased apoptosis. Understanding the direct health implications of climate change is crucial for devising effective strategies to address and mitigate these effects. This study serves as a steppingstone for future research endeavors to explore the direct impacts of climate change-caused conditions on health, identifying risks, vulnerabilities, and uncertainties. Through continued investigations, robust models can be developed to accurately depict how climate change affects community health, paving the way for informed decision-making and adaptation strategies.

## Funding

This work was supported by the Department of Environmental Health Engineering, School of Public Health and Safety, 10.13039/501100022299Shahid Beheshti University of Medical Sciences (Grant number: IR. SBMU.PHNS. REC.1400.114). Author Saeed Motesaddi Zarandi has received research support from 10.13039/501100022299Shahid Beheshti University of Medical Sciences.

## Data availability

Data will be made available on request.

## CRediT authorship contribution statement

**Saeed Motesaddi Zarandi:** Visualization, Validation, Supervision, Methodology, Investigation, Funding acquisition, Formal analysis, Data curation. **Rasoul Yarahmadi:** Visualization, Validation, Methodology, Investigation, Formal analysis, Data curation. **Rasul Nasiri:** Writing – original draft, Visualization, Validation, Software, Methodology, Investigation, Formal analysis, Data curation, Conceptualization. **Mohammad Bayat:** Writing – review & editing, Visualization, Validation, Methodology, Data curation. **Hossein Nasiri:** Writing – review & editing, Visualization, Validation, Methodology, Conceptualization. **Abdollah Amini:** Writing – review & editing, Visualization, Validation, Resources, Methodology. **Mohammad Esmaeil Motlagh:** Resources, Methodology. **Hassan Rasoulzadeh:** Writing – review & editing.

## Declaration of competing interest

The authors declare that they have no known competing financial interests or personal relationships that could have appeared to influence the work reported in this paper.
